# A modeling of complex trait phenotypic variance determinants

**DOI:** 10.1093/pnasnexus/pgae472

**Published:** 2024-10-18

**Authors:** Shobbir Hussain

**Affiliations:** Department of Life Sciences, University of Bath, Claverton Down, Bath BA2 7AY, United Kingdom

## Abstract

Studies have now shown that the heritability of some complex traits, such as human height, can be virtually fully captured via potential use of sufficiently powered approaches that can characterize the associated collective common- and rare-variant additive genetic architecture. However, for other traits, including complex disease traits, full recovery of such narrow sense heritability would still likely fall far short of respective heritability estimates yielded from pedigree-based analyses such as twin studies. Here, it is proposed that such traits could also involve additional types of relevant architecture and underlying genetic mechanism, such that interaction of somatic variants with heritable variants may represent an underappreciated component. The theoretical model suggested predicts that some relevant heritability estimates are systematically inflated by twin studies, and that instead a significant proportion of the phenotypic variances may be explained by specialized types of heritable genotype-by-environment interaction.

## Introduction

Most, if not all, human phenotypic traits can be influenced by both genotype and environment. For a given trait, the proportion of phenotypic variance in a population that is determined by variance in heritable genetic factors is referred to as heritability, with the remaining proportion inferred to be determined by environmental variance (1–3). Pedigree-based analyses such as twin studies can provide estimates of heritability of a phenotypic trait, while experiencing limitations such as that any correlations and interactions present between heritable genotype and environment are difficult to estimate and thus usually ignored and held at zero (4). Twin study analyses otherwise provide good estimates of broad-sense heritability, which is dependent not only on heritable additive contributions, but also on interaction between heritable variants (often referred to as “gene-gene” interactions but more accurately as allele-allele interactions) (4). However, the heritable component of most phenotypic traits in biology is thought to be determined largely, if not virtually exclusively, by additive variance (5–7), and thus twin-study heritability predictions are often taken as close estimates of such narrow-sense heritability too.

More recently, heritability predictions for various traits have also been made using population-level genetic-relatedness studies, referred to as genome-wide complex trait analysis (GCTA), using genotype information captured on single nucleotide polymorphism (SNP) microarrays (8, 9). Such investigations yield predictions of narrow-sense heritability due to common variants in a population, which are sometimes referred to as “SNP heritability” estimates. Genome-wide association studies (GWASs) can be thought of as having aimed to delineate the predicted SNP heritability for a given trait by characterizing the actual genotype-phenotype associations. In what should be considered landmark studies in the human genetics field, this was quite recently achieved comprehensively for human height by Yengo et al. (10), who characterized virtually fully the respective calculated SNP heritability of ∼0.5 predicted for European populations (11). The study mapped ∼7,200 loci, each estimated to span on average ∼90 kb, that likely contain nearly all the causal heritable variants that can determine human height. Furthermore, the associated SNPs within these loci often clustered not only near each other, but also near coding genes already known to play important roles in regulating skeletal growth. Thus, perhaps, the most encouraging finding of the study more generally is support for the “effector gene” concept: the majority of GWAS-identified causal variants generally locate in noncoding DNase I hypersensitive sites, thus suggesting gene regulatory roles (12); here, it is quite possible that the causal variants within loci mapped by Yengo et al. are regulating the expression of the nearby skeletal growth–related effector coding genes. Indeed, recent whole exome sequencing (WES)–based studies to identify rarer coding variants associated with other complex traits have indeed observed a convergence with respective noncoding signals identified by previous relevant GWASs, thus supporting the effector gene concept more generally (13–15).

Further very significant recent developments in the field came via extension of the GCTA principle that enabled estimation of narrow-sense heritability to include rare variants too (in addition to common variants) via relevant analysis of whole genome sequencing (WGS) data (11); such estimates are referred to as “WGS heritability” predictions. Wainschtein et al. (11) used WGS data of ∼25,000 individuals to estimate WGS heritability for height and body mass index (BMI). The estimated WGS heritability for height was ∼0.7 (11), thus almost fully recovering the respective twin-study heritability estimate of ∼0.8 (16). Because the gap between the two estimates was rather modest, it is possible for example that relevant ultrarare variants not detected in the cohort sample used, and structural variants that were not captured by the short-read sequencing, are potential suitable explanations. However, the respective observations for BMI pose a much bigger problem since the estimated WGS heritability was ∼0.3 (11), whereas twin-study heritability estimates stand at ∼0.7 (16–18), thus meaning that additional explanations are most likely required. One possibility is that our assumption that twin-study heritability estimates are representative of mostly/virtually exclusively additive variance is not always correct, and thus interactions between heritable variants (allele-allele interactions owing to epistasis and dominance effects) might for example play a significant role in determining phenotypic variance for some complex traits (4); potential according narrow-sense heritability overestimation has previously been referred to as phantom heritability (19). A second possibility is that interactions between heritable genotype and environment play a significant role, meaning that twin-study heritability estimates themselves would be systematically inflated (4); there is actually some very good evidence in support of this second possibility (18), and this concept will be revisited later on in this article.

It is likely that the aforementioned problems will persist through other types of complex genetic disease too, which also have significantly lower SNP heritability/twin-study heritability ratios compared with that observed for height and to some extent to even BMI. As an example, autism spectrum disorder (ASD) SNP heritability calculated from large-scale studies stands at ∼0.2 (20, 21), while respective twin-study heritability is at ∼0.8 (22). The possibility that such instances may be explained by relatively much more rare variant heritability is looking quite unlikely, because as with both BMI and height, a very recent analysis of a much larger number of complex traits suggested that rare coding variants consistently explain a significant yet much smaller proportion of heritability compared with common variants (23), which is indeed in agreement with the findings of previous studies that analyzed rare vs common variant heritability of expression levels of 414 plasma proteins (24). Furthermore, recent burden heritability regression analyses suggest that rare coding gene variants are likely to explain only just ∼1% to 2% of the phenotypic variance of most complex traits, and that such rare variant heritability was accounted for mostly by ultrarare loss-of-function variants (15). A well-reasoned argument can be made that SNP and WGS heritability estimates, and analogous types of investigation, have for several complex traits analyzed in fact further highlighted the problem of predicted narrow-sense vs twin-study heritability gaps.

As alluded to previously, and as will be expanded on more fully later on in this article, potential roles for heritable genotype-environment interaction as additional significant determinants of phenotypic variance for some complex traits is well worth considering; although such concepts in various capacity have been discussed elsewhere (25), in this article, important relevant roles for somatic mutation are proposed. With regard to noncancerous disease, somatic mutations are sometimes considered as generally occurring too rarely to have more common roles in disease; the validity of such views will be discussed in the following section. It will then be discussed how somatic variants might fit within the currently known architecture of complex disease genetics and how this might enable a more complete understanding of associated genetic mechanisms, thus potentially explaining twin-study heritability inflation while helping recover additional determinants of relevant trait phenotypic variances.

## Somatic mutation, hypermutability, and complex disease

All scholars and students of the biological sciences would be able to tell us that mutation is at the essence of evolution; as conveyed particularly eloquently by Lewis Thomas, “The capacity to blunder slightly is the real marvel of DNA. Without this special attribute, we would still be anaerobic bacteria and there would be no music.” (26). Thus, as opposed to biological flaw that needs to be coped with, mutational capacity may also be viewed as something that has been anciently hardwired into all living cells, consequently including human somatic cells. The human germ line, however, appears to have evolved better means of ensuring genome integrity, and as a matter of fact, the latest single-cell sequencing methods of estimating mutation rates find that somatic mutation rates are almost 2 orders of magnitude higher than in germline cells (27). This probably should not come as too much of a surprise, in fact, considering observed population prevalences of sporadic cancers. For example, the majority of cancer cases in populations are sporadic, and it can be extrapolated from epidemiological studies that at least ∼1 in 4 individuals will develop 1 of such cancers in their lifetime (28). Because such cases of sporadic cancer occur without any family history, this strongly suggests that the occurrence of important function-altering somatic mutation even on noncancerous backgrounds (e.g. the original “driver” mutations) is a very common event. A recent study reanalyzed WGS data from ∼43,000 noncancerous blood genomes collected by the TOPMed consortium (29). Even though the investigation was limited by the fact that only a subset of mutation types were analyzed, along with the fact that the sequence depth was rather low and thus not optimal for somatic mutation calling, the study indeed found that nononcogenic somatic mutation is in fact substantially more common than previously appreciated (29).

A further particularly important consideration is whether mutation rate is more or less uniform across a genome or whether some loci are more prone than others to acquiring sequence variation. For example, it has been theorized that successful protein coding genes might often have originated at hypermutable loci in genomes as this may have enabled more optimal capacity for novel functional gain (30). With time, although negative selection pressures would act to keep mutation rates within protein coding genes low in the population germline, it is still possible that de novo mutations (including both germline and somatic) might be relatively more common, if the genes are in fact hypermutable. Michaelson et al. (30) analyzed WGS data from monozygotic twins and their parents and showed that germline de novo mutation rates can vary by ∼100× across a human genome, and that genes involved in disease were characteristically hypermutable. In general, genes that were paradoxically characterized by hypermutability yet under strong purifying selection were found to much more frequently include disease genes listed in the Online Mendelian Inheritance in Man database. Indeed, the WGS analysis of 10 monozygotic pairs concordant for ASD investigated in the study identified germline de novo mutations in 29 protein-coding genes, 5 of which had been identified as de novo mutated in subjects with ASD while absent in control subjects in previous independent WES studies (30). The authors suggest that hypermutability of genes may be associated with relevant features of their genomic location, such as, for example, nucleosome occupancy and recombination rates. Such an idea is in fact in support of the previous observation that genes likely locate to hypermutable or hypomutable locations of the genome depending on their potential need to adapt (31). Thus, for example, housekeeping genes were found to locate preferentially to hypomutable regions in the genome, whereas genes with more dynamic cellular roles located much more frequently to hypermutable locations (31).

An additional relevant consideration is whether there might exist any significant interindividual differences in somatic mutation rates that may predispose to disease risk. A recent study performed WGS on a total of 131 postmortem brain samples taken from adult neurotypical individuals and adult individuals with ASD, schizophrenia, and Tourette's (32). Samples consisted of ∼1 cm^2^ tissue and were found to typically harbor ∼20 to 60 detectable somatic mutations of the single nucleotide substitution type. Surprisingly, however, ∼6% of the samples harbored hundreds of such type of mutation and the authors refer to these as “hypermutable” samples/brains, although the biological cause of the hypermutability was not clear. In general, the allele frequencies of the single nucleotide substitutions detected were typically in the range of 1% to 10%, suggesting that the cells harboring the somatic mutations were able to undergo significant clonal expansion on nononcogenic backgrounds. Incidentally, 6 putative deleterious somatic mutations in genes previously associated with ASD or schizophrenia were detected in the 131 samples analyzed (32). The observation of differential hypermutability between independent noncancerous individuals is not limited to brain tissue. Olafsson et al. (33) showed that colonic crypts from non-neoplastic Crohn's disease patients often have significantly elevated levels of mutation rate compared with healthy individuals (2.4× for nucleotide substitutions and 7× for indels). Somatic mutations in a number of known inflammatory bowel disease pathogenesis pathway genes were detected, prompting the authors to propose a potentially causal role for these in initiating the disease; this was supported by their observation that clonal expansion of somatically mutated cells occurs to a very significant extent, even in noncancerous adult tissues. Of note, independent colon crypt clones taken from the same inflammatory bowel disease patient often harbored distinct somatic mutations in the same gene, leading the authors to speculate that the selection and clonal expansion of cells harboring distinct somatically mutated genes within colon microenvironments may be influenced by an individual's germline genetic background (33, 34). Earlier crucial studies had also established the roles of somatic variants in clonal hematopoiesis and risk for cardiovascular disease and other age-related conditions (35–39), as well as their potential relevant interplay with germline variants, and such observations have been well reviewed elsewhere (40, 41). Related findings include those from a number of studies showing that somatic mutation harboring cells undergoing significant clonal expansion is in fact a pervasive phenomenon in various normal tissue types (42, 43).

Although studies specifically aiming to identify somatic mutation in other complex genetic diseases are quite rare in the literature, a few further examples stand out. Studies have aimed to identify somatic mutations associated with ASD by primarily analyzing peripheral samples such as blood and saliva (44, 45). Thus, although limited by the fact that only relevant somatic mutations occurring potentially during early development would be detectable, the investigations uncovered significant enrichment of such mutation in individuals with ASD compared with control subjects; the variants very frequently included nonsynonymous mutation occurring in protein coding sequence (44) as well as large copy number variations (45) in previously established ASD-associated genes/loci. Another study collected 55 postmortem ASD brains and performed sequencing at a depth of ∼500× on 78 preselected ASD-associated protein coding genes from dissected brain samples (46), thus enabling relevant somatic mutations occurring post–embryonic development to be potentially detected too. The study found a very significant enrichment of somatic mutation in the 78 candidate genes tested in the 55 brains of subjects with ASD compared with 50 control brains, including 6 loss-of-function mutations in ASD samples vs zero loss-of-function mutations in control subjects (46). A similar study has also performed relevant analysis to assess the association of somatic mutation with Alzheimer's disease by performing high-coverage (∼600×) WES from hippocampal formation tissue from 52 patients with Alzheimer's (47). Using matched blood sample sequencing, 12 (23%) of the patients were found to harbor likely pathogenic germline mutations. From the hippocampal tissue samples analyzed, 14 (27%) of the patients were found to harbor putative pathogenic somatic mutations, which were protein structure–altering variants in genes involved in pathways affecting tau phosphorylation, which is key mediator of Alzheimer's pathogenesis. An important and general point that is apparent from the preceding discussions is that while anatomical location of a somatic mutation is a central consideration, the timing of the mutation would also be a key determinant of its potential phenotypic impact. If we take a very simple example, a relevant mutation arising in the hippocampus later in life might have the potential to contribute to Alzheimer's risk but would have negligible impact on ASD risk, even though both disorders might at least in part be associated with similar pathways of hippocampal dysfunction (48–50). This can have an important bearing on relevant future study designs of developmental disease in which for example sampling of blood or saliva for detection of associated potential early developmental somatic mutation may offer advantages (44, 45).

Given relevant observations in recent years that have been largely aided by technological advances in DNA sequencing, it is arguably likely, or at the least very conceivable, that somatic mutation plays a much more common role in noncancerous diseases than previously thought. However, such investigations have thus far been few and far between and where performed have mostly involved only very small sample sizes; the identification (unlike in cancer, disease-affected tissue is not always readily anatomically identifiable), and availability, of disease-affected tissue is a major hindrance in the field, and this trend is likely to continue unless there might be some concerted effort for much larger-scale collection of such samples.

## Somatic interaction variance in phenotypic variance determination

Our usual definition of somatic variation may involve that it is genetic. However, in the context of our discussions here, this would not be correct because they are nonheritable; somatic variants are thus more accurately viewed as a component of the environment. Nonetheless, interaction of such environmental covariates with heritable genotype is possible, and for example, if such interactions are present but ignored could result in relevant twin-study heritability estimates being inflated (elaborated on later in this section). A closer look at more general previous knowledge of heritable genotype-environment interactions in complex trait phenotypic variance determination is thus relevant. Perhaps the most compelling observations on this front have been made by Robinson et al. (18) during relevant studies of BMI. It was demonstrated that, across common SNP loci, heritable genotype-environment interactions such as genotype-age and genotype-smoking habit can make significant contributions (8% and 4%, respectively) to the population phenotypic variance. The study found no evidence of heritable genotype-covariate interaction for an additional 7 environmental variables investigated, although the study was limited by the fact that it was only sufficiently powered to detect relevant interactions that contributed at least 4% of BMI phenotypic variance. Another study indeed suggested that heritable genotype-environment interactions may in fact occur pervasively in contributing to population BMI phenotypic variation (51).

The majority of heritable variants that contribute to polygenic complex trait phenotypic variances are thought to be inherited in the heterozygous state. These most importantly include noncoding common variants of low penetrance, which can presumably influence the expression of nearby effector coding genes, or rarer variants of relatively larger penetrance located within coding genes themselves. We may imagine a scenario in which a risk variant within a coding gene is inherited in the heterozygous state and thus is only weakly, if at all, phenotypically expressed, but that disruption of the second copy of the gene then results in much stronger associated phenotypic expression; indeed, extrapolation from Mendelian genetics raises the possibility that this may well be a fairly common occurrence. Accordingly, phenotypic expression of a relevant heritable variant could be significantly amplified/rendered by a somatic mutation occurring on the second copy of the coding gene; this would represent an example of a heritable genotype-environment interaction. Perhaps more importantly, this principle can be extended to include inherited noncoding variants: these may, for example, reduce the expression of one copy of an associated effector coding gene in cis with only very weak, or even zero, phenotypic effect, but such effect may be amplified/rendered by a somatic mutation within the second copy of the effector gene; this again would equate to a heritable genotype-environment interaction. A prediction of such a model is that heritable variants that are dependent on somatic mutation for phenotypic expression may generally be associated with lower calculated population effect sizes compared with those that are not. Furthermore, dependent heritable variants linked with relatively more mutable genes may be predicted to display a trend toward being associated with higher calculated population effect sizes compared with dependent heritable variants linked with less mutable genes. An additional important note is that although inherited high-penetrance coding gene mutations are only very rarely observed, owing to purifying selection, virtually no such selection pressures exist on somatic high-penetrance coding mutations, and these thus conceivably may be more frequently occurring in populations, raising the possibility of more significant impacts on polygenic complex trait phenotypic variances.

New mutation is not a completely random process, in fact in complex biological systems, it is probably far from it. Indeed, mutation rates and mutation spectrum are found to vary significantly between human populations (52, 53), and rates of new mutation can also vary significantly between families (54–56). Mutation rate and spectra are known to be influenced by genetic variants, sometimes referred to as “mutators” or “antimutators,” for example associated with DNA repair genes. However, other genetic factors, for instance, those that regulate the subtle balance of cellular dNTP pools, can also act as mutators (57, 58). Even much less obvious examples of mutators and antimutators also probably exist; for example, a multitude of cellular metabolic pathways can produce endogenous mutagens capable of inflicting DNA insults (59, 60), and potential genetic variants associated with such pathways would also have the means to influence rates and spectrum of mutation. A further relevant consideration is the fact that endogenous genetic events, such as somatic LINE-1 gene retrotransposition for example, can also be mutagenic and have previously been shown to be a significant source of genomic sequence variation within the mammalian brain (61–63). Collectively, such observations suggest that rate of new mutation is likely to be, at least in part, a heritable entity. Incidentally, GWASs for complex disease commonly suggest pleiotropic effects in which the same heritable variants are often associated with otherwise clinically distinct phenotypes (64); one could speculate that such heritable variants might also include those that influence rates and spectrum of new mutations. Under the model proposed here, for example, such “secondary” heritable variants may influence the likelihood of types of somatic mutations that themselves may be capable of interacting with relevant heritable variants more directly linked to associated disease pathways (as discussed in the preceding paragraph). Also, as alluded to previously, somatic cells in particular appear to be much less efficient in avoiding/repairing DNA damage compared with the germline, given the substantially higher rates of mutation observed (27), and therefore may be much more susceptible to the relevant effects of mutator and antimutator alleles. A related but distinct consideration is whether flanking DNA sequence context and local genomic features may influence the likelihood of a somatic mutation occurring at a specific site, as studies have shown that such factors have a significant effect in determining the likelihood of new mutations (65–67).

Undoubtedly, environmental factors, such as ageing and mutagen intake/exposure, would increase the opportunity and means respectively for somatic mutation and are therefore also relevant considerations. Examples of mutagen intake, for example, may include smoking habit, especially because this may entail systemic exposure due to associated mutagens entering the bloodstream (68, 69). Indeed, classic examples of relevant genetic alterations detectable in blood, such as mosaic loss of the Y chromosome, which displays significant heritability (70, 71), as well as other genetic variations, have previously been significantly associated with ageing and smoking status (72) and may for example increase susceptibility to diseases such as Alzheimer's (37).

A hypothetical modeling of how the independent concepts discussed in this section may collectively contribute to determining phenotypic variance of relevant complex traits, and how this may be associated with twin-study heritability estimate inflation, is presented in Box 1.

Box 1. **Hypothetical modeling of somatic variant interactions in phenotypic variance partitioning**

Phenotypic variance is classically partitioned as

(1) $$V_{P}=V_{G}+V_{E}$$

where V_P_ represents phenotypic variance, V_G_ denotes total heritable variance (summing heritable additive variance, heritable epistasis variance, and heritable dominance effect variance), and V_E_ denotes environmental variance. Most relevant applications usually assume that heritable genotype-environment correlations and interactions are small or negligible and can thus be ignored. Nonetheless, a more complete partitioning is given as

(2) $$V_{P}=V_{G}+V_{E}+2\mathrm{co}v_{\mathrm{GE}}+V_{\mathrm{GxE}}$$

where the covariance term cov_GE_ refers to correlation between heritable genotype and environment (for example, in which a “favorable” genotype yields a “better” phenotype that may in turn nurture a favorable environment, thus improving the phenotype even further; such correlation effects are not so relevant to the discussions presented here, but the keen reader is referred to Falconer) (4). The component V_GxE_ refers to heritable genotype-by-environment interactions (in which both genotype and environment can modify the effects of each other) and is much more relevant here. If V_GxE_ effects are present but ignored, this will lead to estimates of V_E_ being inflated, when considering that the source of the variation is environmental; however, if the source of variation is genetic, it follows that this will lead to estimates of V_G_ being inflated. The previous has been adapted from previous scholarly work by Falconer (4).

Somatic variants are nonheritable and are thus a component of the environment. In the model outlined in this article, it is suggested that somatic variants can modify the expression of heritable variants, and such instances would thus represent examples of GxE effects. We may therefore further partition V_GxE_ as

(3) $$V_{\mathrm{GxE}}=V_{\mathrm{GxX}}+V_{\mathrm{GxS}}$$

where V_GxX_ represents heritable genotype-by-extrinsic-environment interactions, and V_GxS_ denotes heritable genotype-by-somatic-variant interactions. The occurrence of somatic mutations is influenced by heritable genetic factors as well as by environmental factors. Thus, V_GxS_ itself may be further partitioned as such:

(4) $$V_{\mathrm{GxS}}=V_{\mathrm{GxSxM}}+V_{\mathrm{GxSxX}}$$

where V_GxSxM_ involves instances where the occurrence of somatic mutation is influenced by heritable variants (such as, for example, mutator/antimutator variants, flanking sequence variants, etc.) and is an example of a GxExG interaction. V_GxSxX_ involves instances in which the occurrence of somatic mutation is influenced by extrinsic environmental factors (such as, for example, aging and mutagen intake/exposure) and is an example of a GxExE interaction. This would yield a relatively more complete partitioning as

(5) $$V_{P}=V_{G}+V_{E}+2\mathrm{co}v_{\mathrm{GE}}+V_{\mathrm{GxX}}+V_{\mathrm{GxSxM}}+V_{\mathrm{GxSxX}}$$

For some complex traits, the theoretical model outlined in this article predicts that the values of the V_GxS_ components in [3] and [4], and thus the value of the V_GxE_ component in [3] and [2], would in fact be significant, and thus if ignored would lead to estimates of V_E_ and V_G_ collectively being significantly inflated because the sources of variation relevant to the interactions described are both environmental and genetic. The predicted systematic inflation of the V_G_ component would lead to relevant twin-study heritability estimates, H^2^, defined as the ratio V_G_/V_P_, being overestimated.

A final point perhaps worth of brief mention here is regarding reports that somatic mutation rates can vary between males and females, sometimes in a locus-specific manner, with males more generally displaying higher rates but with females displaying elevated rates within at least some loci (73–77). However, such observations are thus far only sparsely characterized, and whether relevant principles might have any bearing on helping explain the sex dimorphism observed (where one particular sex is much more frequently affected than the other) for some complex disease traits would at this stage be only very speculative.

## Relevant future investigation

Much of the early frustration in the human genetics field relating to outputs from GWAS investigations stemmed from the fact that the risk variants identified were generally only of much smaller effect sizes than originally anticipated, and that the majority were located in noncoding regions of the genome while offering little clue regarding their relevant biological effects (78). A reasonable counterargument is that GWASs are unbiased approaches that promise to characterize highly polygenic genotype-phenotype correlations, at the least to enable better risk predictions and patient stratification within a population; they make no guarantees of illuminating disease mechanism. Thus, while in many regards GWASs can be viewed as having even gone beyond fulfilling their promise (79), the aforementioned issues still persist throughout much of the field. Accordingly, although GWASs still have important roles to play, as exemplified by the recent high-powered studies of human height (10), this has driven some in the field to focus more on WES-based investigations to identify rarer coding variants that may more readily help yield clues on relevant disease mechanisms (15, 80, 81). Such studies may also be further particularly useful, as they also have the potential to help identify the effector genes that may be under the regulation of noncoding regulatory elements found to be associated with complex traits by GWAS investigations (13–15). An independent perhaps notable finding emerging from such WES-based investigations is that cases of complex disease in some individuals may be caused by highly penetrant mutation of even just a single gene, prompting the suggestion of a continuous spectrum of monogenic to polygenic mode of complex trait determination (80). However, whether individual complex disease traits might, at least to some degree, include aggregates of single-gene disorders remains debatable, and such a concept may be much more relevant to more clearly dichotomous complex traits such as for example relevant congenital disease (82). In any case, more comprehensive identification of complex trait–associated coding genes, including effector genes, via much larger scale WES-based investigations should in addition represent a major focus of future study in the field.

The previous discussion is relevant to potential investigation of the model proposed in this article because identification of complex trait–associated coding genes (including effector genes) will also be very helpful in investigating potential roles of relevant somatic mutation. Detection of somatic mutations in previously established disease-associated coding genes within affected individuals would indicate a role in disease risk; however, because most complex disease traits are likely highly polygenic, WES to survey for somatic mutations across the whole exome would still be appropriate and useful. Somatic mutation screening would most ideally be performed in affected tissue, if such tissue is readily identifiable and available. Otherwise, more routinely acquired samples, such as blood or buccal samples, may be used, but such a strategy would be limited to the detection of relevant mutations potentially occurring during early development. Such investigations could be performed via appropriately customized analyses of available relevant population cohort genome sequencing data, but analysis of sequence data from monozygotic twins both concordant and nonconcordant for the disease under study would also be very useful. Both twins of disease-affected concordant pairs should relatively more frequently exhibit concordance for somatic mutation, ideally in established disease-associated coding genes, although not necessarily in the same genes, compared with twins of phenotypically nonconcordant pairs. For phenotypically nonconcordant pairs, higher frequency of somatic mutation in established disease-associated coding genes would be expected in the affected twin compared with that observed in the unaffected twin.

## Summary

Phenotypic variances of most complex traits in biology are likely determined largely by additive genetic variance, but it is possible that some, including for complex diseases, may also be significantly contingent upon specialized types of heritable genotype-environment interaction variance as outlined in this article. A prediction of the model is that complex traits that are largely explained by additive genetic variance may involve coding genes (including effector genes) that are much more frequently located at nonhypermutable genomic loci, whereas those found to be only partially explained by additive variance could potentially involve significant numbers of the associated genes located at hypermutable loci.

A number of different approaches are currently used and have been suggested to identify further variants that make up the genetic architecture of complex disease (79, 83); such efforts are likely to improve our understanding of the relevant disorders further. Nonetheless, a potentially more complete understanding of the underlying genetic mechanisms may be just as important. It may be useful to consider additional relevant theoretical models that are nonetheless compatible with empirical observations in the field, such as for example the one proposed here.

## Data Availability

There are no data underlying this work.
